# Supplementary material for: Photonic time-crystalline behaviour mediated by phonon squeezing in Ta2NiSe5
Source: Nat Commun. 2024 Apr 29;15:3638. doi: 10.1038/s41467-024-47855-8 (PMC11059354; doi:10.1038/s41467-024-47855-8)
Supplement: Supplementary file 1 — Supplementary Information [file 41467_2024_47855_MOESM1_ESM.pdf]

Supplementary Information to "Photonic time-crystalline behaviour mediated by phonon squeezing in Ta_2NiSe_5"

Marios H. Michael,[1,2,*] Sheikh Rubaiat Ul Haque,[3,4,†] Lukas Windgaetter,[2] Simone Latini,[2] Yuan Zhang,[3] Angel Rubio,[2,5] Richard D. Averitt,[3] and Eugene Demler[1,6]

[1]*Department of Physics, Harvard University, Cambridge, Massachusetts 02138, USA*

[2]*Max Planck Institute for the Structure and Dynamics of Matter, Luruper Chausse 149, 22761 Hamburg, Germany*

[3]*Department of Physics, University of California San Diego, La Jolla, California 92093, USA*

[4]*Stanford Institute for Materials and Energy Sciences, SLAC National Accelerator Laboratory, Menlo Park, CA 94025, USA*

[5]*Center for Computational Quantum Physics, The Flatiron Institute, 162 Fifth Avenue, New York, 10010 New York, USA*

[6]*Institute for Theoretical Physics, ETH Zürich, 8093 Zürich, Switzerland.*

(Dated: March 27, 2024)

Phonon	Symmetry	Frequency (THz)
21	B_u	4.238
22	A_u	4.314
25	A_u	4.634
26	B_u	4.699

Supplementary Table I: IR-active phonon modes with a frequency close to 4.5 THz.

Supplementary Note 1

In this note we present the DFT calculations of IR-phonons in Ta_2NiSe_5. The phonon spectrum at Γ has been computed using the Density Functional Perturbation Theory routines of the VASP code [1–4] using the vdW-opt88 functional [5, 6] on a 48x4x6 mesh. The Born effective charges have been computed on a similar k-mesh, the IR-activity has been computed using phonopy [7] and the phonon spectroscopy code by Skelton et. al [8]. The electronic bandstructure plots have been obtained using a 24x4x6 k-mesh and a 320 eV cutoff using the standard PBE functional.

1. *Phonons*

For the monoclinic geometry there are 21 symmetry allowed IR-active modes, 11 B_u and 10 A_u modes.Of these modes, only four are in a frequency range near 4.5THz, listed in Supplementary Table I.

In the main text we identify phonon 26 which has a frequency of 4.699 THz as the relevant for phonon for parametric amplification. Therefore, we have investigated how this phonon evolves across the structural phase transition. To do this we have linearly interpolated between the monoclinic and high temperature orthorhombic atomic and lattice geometry using an interpolation parameter d.

$$\begin{aligned} \vec{v}_{\mathrm{i}}(d) &= \vec{v}_{\mathrm{o}} + d \cdot \vec{v}_{\mathrm{t}} \\ \vec{v}_{\mathrm{lat,i}}(d) &= \vec{v}_{\mathrm{lat,o}} + d \cdot \vec{v}_{\mathrm{lat,t}} \quad , \end{aligned} \tag{1}$$

* Correspondence to: marios.michael@mpsd.mpg.de
† Correspondence to: rubaiath@stanford.edu

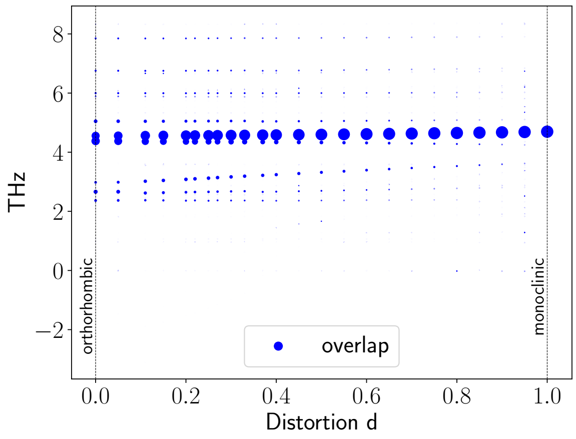

Supplementary Figure 1: Overlap of the 4.7 THz monoclinic phonon with the phonons calculated along the orthorhombic to monoclinic phase transition. The dot size is proportional to the overlap. The figure shows that the 4.7 THz phonon predominantly hybridizes into two orthorhombic phonons at frequencies of 4.38 THz and 4.56 THz. Note that the orthorhombic structure exhibits two phonon instabilities with complex frequency which are displayed as negative energies here.

with the transition vector for the atomic configuration and the lattice defined as

$$\begin{aligned} \vec{v}_{\mathrm{t}} &= \vec{v}_{\mathrm{m}} - \vec{v}_{\mathrm{o}} \\ \vec{v}_{\mathrm{lat,t}} &= \vec{v}_{\mathrm{lat,m}} - \vec{v}_{\mathrm{lat,o}} \quad . \end{aligned} \tag{2}$$

The subscripts m denotes the monoclinic geometry and o the orthorhombic geometry. Because the phonon eigenmodes form an orthonormal basis for the movement of all atoms we can trace how the monoclinic 4.7 THz phonon hybridizes along the phase transition into the orthorhombic phonons, by calculating the overlap of the monoclinic phonon eigenvector with the phonon eigenvectors along the transition. The result is shown in figure 1. It shows that the 4.7 THz phonon predominantly disperses into two orthorhombic phonons at 4.38 THz and 4.56 THz.

2. *Frozen phonon calculations*

To estimate the electron phonon coupling of the four IR-active phonon modes displayed in Supplementary Table I, we show frozen phonon band structure calculations for these modes. As discussed in the main text, only the strongly amplified IR mode should display a con-

	Phonon21 (meV)	Phonon22 (meV)	Phonon25 (meV)	Phonon26 (meV)
cond. Band 1	1.197	2.762	3.321	8.171
cond. Band 2	1.568	2.378	1.324	24.953
cond. Band 3	5.363	3.391	6.473	34.436
cond. Band 4	1.329	5.657	17.629	11.814

Supplementary Table II: Coupling as defined by equation (3) for the bandstructure presented in figure 2 . Only states near the band-edge along Z-Γ have been considered for the summation in Supplementary Eq. (3). One can see that conduction bands 2 and 3 shift in opposite directions upon displacing along phonon mode 26 (see Supplementary Table I). This is consistent with the parametric driving mechanism presented in the main text.

siderable coupling to the electronic bands. Upon displacing the atomic positions along the eigendisplacement of the four phonon modes and recalculating the electronic bandstructure, only mode 26 shows a strong modulation of the conduction bands, indicating a strong coupling of this mode to the electronic degrees of freedom upon excitation (see Supplementary Fig. 2).

To quantify the strength of the electron-phonon coupling λ_i, we define

$$\lambda_i = \sum_k w_k |\ (E_{i,k})\big|_{\langle Q\rangle=0} - (E_{i,k})\big|_{\langle Q\rangle\neq 0}\ |, \tag{3}$$

as the difference of the Kohn-Sham energies at the atomic equilibrium position $\langle Q\rangle = 0$ and the frozen phonon displaced position $\langle Q\rangle \neq 0$. k runs over all k-Points along the chosen Z-G path near the bandedge and w_k is the symmetry weight of the k-Point. Computing this coupling for the band structures shown in figure 2 one obtains for the first four conduction bands the values presented in Supplementary Table II. It shows that the strongest coupling of the conduction bands is to phonon mode 26. For this mode the second and third conduction band are shifted into opposite directions along the Z-Γ near the band edge which is consistent with the above proposed two band model. Therefore, we identify this mode as the relevant mode for the parametric amplification process.

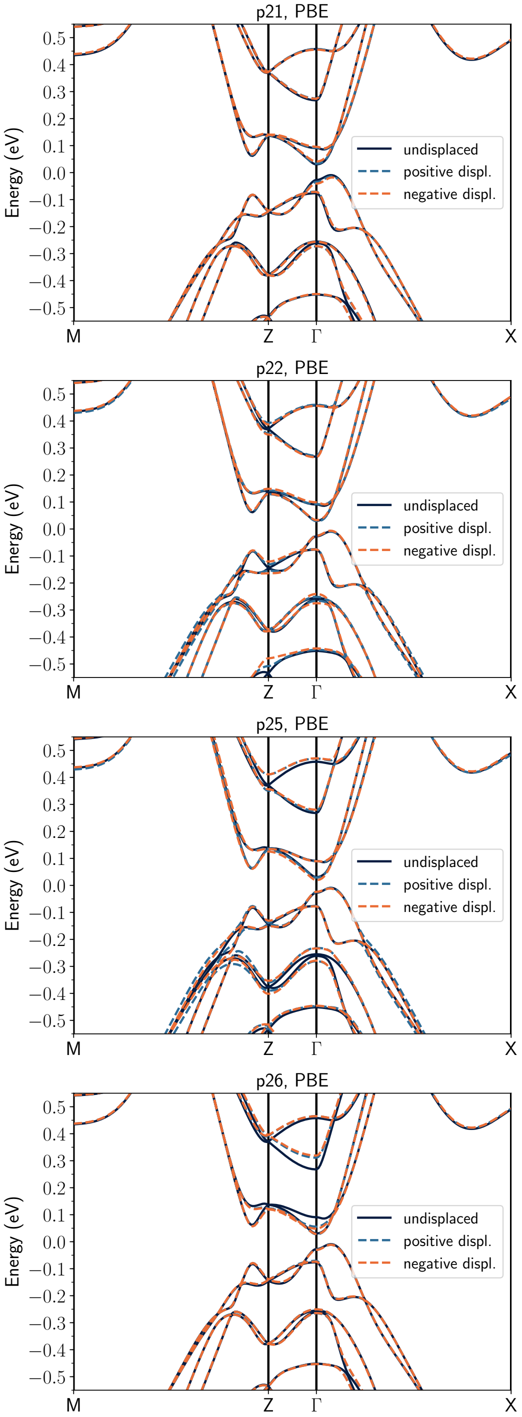

Supplementary Figure 2: Frozen phonon bandstructure of all possible IR active active phonons in the monoclinic phase between 4.2 THz and 4.7 THz using the PBE functional. Phonon 26 shows she strongest coupling to the bandstructure upon displacement along its eigenmode. Most importantly, the second and third conduction band are modulated by this mode and are displaced in opposite directions.

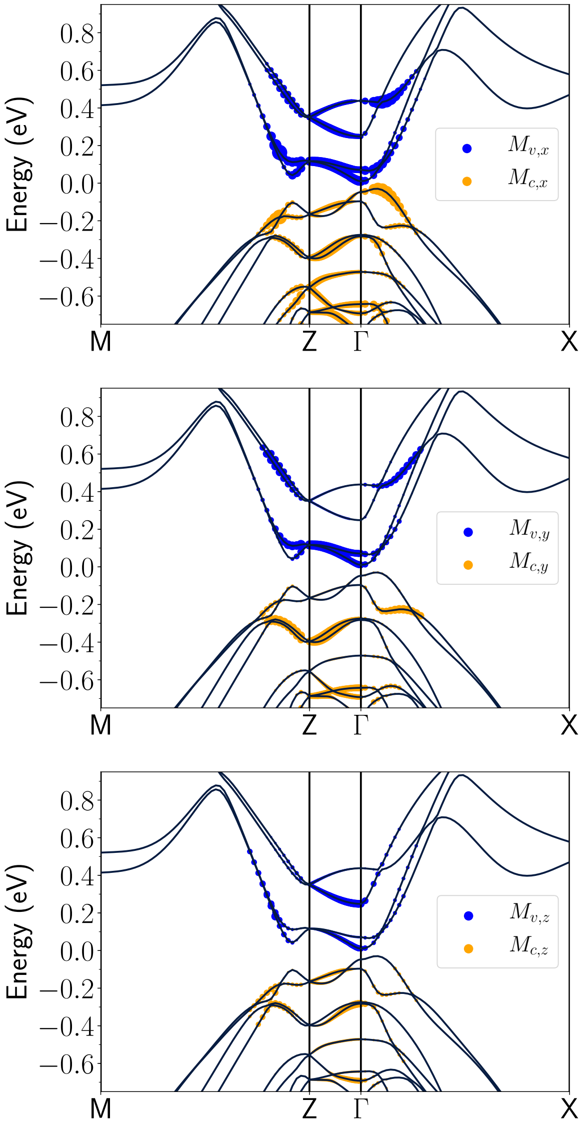

Supplementary Figure 3: Calculation of the dipole matrix elements for all three cartesian directions. We filtered only transitions in the experimentally relevant energy range between 0.33 eV and 0.9 eV. The size of the dots shows the size of the dipole matrix element at a given k-Point and band.

3. Optical transitions

In the experiment, parametric amplification has been pumped with a 0.5 eV laser. In the above section we have identified a phonon mode which couples strongly to the second and third conduction band and might be responsible for the parametric amplification process. To fully justify this claim we need to show that using the 0.5 eV pump electrons are indeed

excited into these strongly coupled conduction band states. Here we have computed the optical transition matrix elements and plotted them as dots on the bandstructure, where the dot size for the valence and conduction bands corresponds to

$$\begin{aligned} M_{\lambda,v,k} &= \sum_c M_{\lambda,v,c,k} \\ M_{\lambda,c,k} &= \sum_v M_{\lambda,v,c,k} \end{aligned} \tag{4}$$

with $M_{\lambda,v,c,k} = |\langle\phi_{v,k}|(-i)\frac{d}{dk_\lambda}|\phi_{c,k}\rangle|^2$. k labels the k-point, v the valence, c the conduction band involved in the optical transition and λ the Cartesian directions.

The result for all three Cartesian directions is shown in figure 3. We have have filtered only the experimentally relevant transitions between 0.33 eV and 0.9 eV transition energy. Such a large energy window should compensate for any underestimation of the electronic gap given by the PBE functional. One sees that optical transitions in x-directions are strongest for the first 3 conduction band states. Thus, we can conclude that upon pumping with 0.5 eV there are many electrons excited into the electronic states that couple strongly to phonon 26 and can lead to the parametric amplification process.

---

[1] G. Kresse and J. Hafner, Ab initio molecular dynamics for liquid metals, Phys. Rev. B **47**, 558 (1993).

[2] G. Kresse and J. Furthmüller, Efficient iterative schemes for ab initio total-energy calculations using a plane-wave basis set, Phys. Rev. B **54**, 11169 (1996).

[3] G. Kresse and J. Furthmüller, Efficiency of ab-initio total energy calculations for metals and semiconductors using a plane-wave basis set, Computational Materials Science **6**, 15 (1996).

[4] G. Kresse and J. Hafner, Norm-conserving and ultrasoft pseudopotentials for first-row and transition elements, Journal of Physics: Condensed Matter **6**, 8245 (1994).

[5] J. Klimeš, D. R. Bowler, and A. Michaelides, Van der waals density functionals applied to solids, Phys. Rev. B **83**, 195131 (2011).

[6] J. Klimeš, D. R. Bowler, and A. Michaelides, Chemical accuracy for the van der waals density functional, Journal of Physics: Condensed Matter **22**, 022201 (2009).

[7] A. Togo and I. Tanaka, First principles phonon calculations in materials science, Scripta Materialia **108**, 1 (2015).

[8] J. M. Skelton, L. A. Burton, A. J. Jackson, F. Oba, S. C. Parker, and A. Walsh, Lattice dynamics of the tin sulphides SnS_2, SnS and Sn_2S_3: vibrational spectra and thermal transport, Phys. Chem. Chem. Phys. **19**, 12452 (2017).
